# Longitudinal assessment of blood-borne musculoskeletal disease biomarkers in the DE50-MD dog model of Duchenne muscular dystrophy

**DOI:** 10.12688/wellcomeopenres.17398.2

**Published:** 2022-08-17

**Authors:** Dominique O. Riddell, John C. W. Hildyard, Rachel C. M. Harron, Dominic J. Wells, Richard J. Piercy

**Affiliations:** 1 Comparative Neuromuscular Diseases Laboratory, Department of Clinical Science and Services, Royal Veterinary College, London, NW10TU, UK; 2 Department of Comparative Biomedical Sciences, Royal Veterinary College, London, NW10TU, UK

**Keywords:** Dystrophin, DMD, biomarker, blood, serum, DE50-MD, canine

## Abstract

**Background:** Duchenne muscular dystrophy (DMD) is a fatal muscle wasting disease caused by mutations in the dystrophin gene. Due to their phenotypic similarity to human patients, large animal models are invaluable tools for pre-clinical trials. The DE50-MD dog is a relatively new model of DMD, and carries a therapeutically-tractable mutation lying within the hotspot for human patients, making it especially valuable. Prior to conducting therapeutic trials using this novel animal model, it is essential to establish a panel of viable biomarkers.

**Methods:** We evaluated a panel of blood-borne biomarkers of musculoskeletal disease in the DE50-MD dog. Venous blood samples were obtained monthly throughout an 18-month study period in DE50-MD (N=18) and wild-type (WT) control (N=14) dogs. A panel of potential plasma/serum biomarkers of DMD was measured and their theoretical utility in future clinical trials determined using sample size calculations.

**Results:** Compared to WT dogs, DE50-MD dogs had substantially higher circulating creatine kinase (CK) activities, myomesin-3 (MYOM3), and the dystromiRs miR-1, miR-133a and miR-206, but significantly lower serum myostatin concentrations. An age-associated pattern, similar to that observed in DMD patients, was seen for CK and MYOM3. Sample size calculations suggested that low cohort sizes (N≤3) could be used to detect up to a 50% improvement in DE50-MD results towards WT levels for each biomarker or a combination thereof (via principal component analysis); as few as N=3 animals should enable detection of a 25% improvement using a combined biomarker approach (alpha 0.05, power 0.8).

**Conclusions:** We have established a panel of blood-borne biomarkers that could be used to monitor musculoskeletal disease or response to a therapeutic intervention in the DE50-MD dog using low numbers of animals. The blood biomarker profile closely mimics that of DMD patients, supporting the hypothesis that this DMD model would be suitable for use in pre-clinical trials.

## Introduction

Duchenne muscular dystrophy (DMD) is a fatal, X-linked muscle wasting disease that affects approximately 1 in 6000 human male births worldwide ^1^. It is caused by mutations in the dystrophin gene that lead to an absence of functional dystrophin protein, a protein crucial to the structural integrity of muscle as well as having important roles in muscle cell signalling ^2^. DMD symptoms include progressive muscle weakness due to repeated cycles of muscle degeneration/regeneration and eventual replacement of muscle with non-contractile tissue ^3^. DMD patients become wheelchair-bound in their early teenage years and affected individuals die most commonly as a result of respiratory weakness and infection, or heart failure in their third or fourth decade of life ^4, 5^. There is currently no cure for DMD and there is a need for more clinically relevant animal models of the disease.

The DE50-MD dog, a recently established canine model of Duchenne muscular dystrophy (DMD), has a point mutation in the 5’ donor splice site of intron 50 that results in deletion of exon 50 from dystrophin gene transcripts, with concomitant frameshift and premature protein truncation ^6^. This mutation lies within the human mutational “hotspot” region (exons 45-53) and is amenable to exon 51 skipping therapeutic approaches, which would be applicable to the largest proportion of DMD patients ^6, 7^. In a recent collaboration, we used the DE50-MD model to demonstrate the first successful use of systemic CRISPR/Cas9-mediated gene editing for DMD in a large animal ^8^.

For extended therapeutic trials using this novel animal model, it is essential to establish a panel of viable biomarkers. Unlike diagnostic biomarkers (which predominantly serve to distinguish healthy from diseased samples), these biomarkers should not only accurately differentiate diseased from normal animals, but their variance should also be low, so that there can be objective quantitation of therapeutic effect size – the degree to which the disease phenotype is ameliorated by specific treatments. Markers exhibiting modest but consistent changes might therefore prove more useful than those with dramatic but variable differences. Historically, biomarkers commonly used in DMD include indices of motor, respiratory or cardiac function, muscle magnetic resonance imaging (MRI), immunohistochemistry (IHC) of muscle biopsy samples, and blood-borne molecules.

Several urinary biomarkers of DMD have been identified, offering a non-invasive biomarker option ^9– 11^. However, compared to blood, the urine biomarker profile of DMD is currently not as well established in animal models or human patients, with a limited number of validated biomarkers of dystropathology ^12^. Further, for practical reasons, obtaining free catch urine on demand from animal models can also be very challenging. For these reasons, we chose not to utilise urinary biomarkers for the current work. In contrast, blood biomarkers are particularly attractive for extended trials: unlike muscle biopsy, blood sampling can be performed comparatively frequently, and samples can be obtained quickly and minimally invasively (without recourse to anaesthesia), at only modest expense. Quantification of creatine kinase (CK) activity, released from the sarcoplasm into the extracellular space when muscle fibres are damaged, is currently the most commonly used blood biomarker for DMD ^13^: its serum and plasma activity is markedly higher in DMD patients than healthy controls ^14, 15^, making it a valuable diagnostic screening tool. Serum CK activity is widely used in the golden retriever muscular dystrophy (GRMD) and canine X-linked muscular dystrophy in Japan (CXMDJ) models at all ages, as it is elevated from as early as one hour of age in comparison with controls ^16– 18^. Serum CK activities are highly sensitive to muscular exertion in DMD, however, and can consequently vary by orders of magnitude between individuals (or between samplings from the same individual), potentially limiting the utility of this biomarker in assessing relative disease severity or subtle improvements ^19, 20^. Further, large elevations in CK activity arise from even modest degrees of muscle damage ^21^. These limitations were revealed in a gene therapy study using the GRMD canine model where prominent and sustained reductions in serum CK activity were not recognised in treated animals, despite improvements in other phenotypic features ^22^.

Potentially a more useful serum biomarker could be the myofibrillar structural protein myomesin-3 (MYOM3). MYOM3 is found at the M-band of the sarcomere in striated muscles, and functions in sarcomere stability during stretching ^23^. As with CK, MYOM3 can be released into the extracellular space following muscle damage, and accordingly, elevated serum concentrations of this protein are found in human DMD patients, *mdx* mice and the GRMD canine model of DMD ^24^. MYOM3 concentrations in DMD patients and *mdx* mice correlate with muscle mass, and thus follow a similar pattern to serum CK activity with age (being higher in young DMD patients compared to healthy controls and older DMD patients ^24, 25^), but this marker exhibits less inter-individual variation ^24^. A study by Rouillon *et al.*, 2015, showed that MYOM3 serum concentrations in the GRMD dog model were 100 times higher in affected compared to control dogs ^24^, though only wild type (WT) and GRMD dogs at 2 and 18 months of age were compared. Serum MYOM3 concentrations were also reported to inversely correlate with extent of muscle dystrophin restoration following exon skipping in *mdx* mice, revealing its use as a biomarker for pre-clinical treatment trials ^24^.

Another protein that has long been of interest in DMD, but has only recently gained attention as a biomarker, is myostatin (MSTN), a protein within the transforming growth factor β family that is secreted by skeletal muscle to modulate muscle growth ^26^. Loss of one or both copies of the MSTN gene results in profound systemic muscle hypertrophy ^27, 28^, and thus promotion of muscle growth via MSTN inhibition has been investigated as a potential therapy for DMD. However, despite promising results in pre-clinical mouse studies ^29– 31^, clinical trials have failed to produce sufficient evidence of clinical efficacy in humans ^32– 34^. One proposed explanation for the lack of efficacy is that patients with muscular dystrophy already have very low levels of MSTN compared to healthy subjects ^35^. Indeed, several studies have found reduced concentrations of circulating MSTN in DMD patients ^31, 35– 37^, as well as in the GRMD dog model of DMD ^38^. Further, reduced mRNA expression of MSTN and its receptor (activin receptor IIB, ACVRIIB) in muscle biopsy samples from both DMD patients and GRMD have been reported, suggesting a downregulation of MSTN pathways ^35, 38^.

In addition to these proteins, circulatory microRNAs (miRs) represent promising potential candidates for serum biomarkers: miRs can be isolated from small volumes (100-200 ul) of serum comparatively easily, and following reverse transcription and quantitative polymerase chain reaction (RT-qPCR) miRs can be detected with very high specificity, and a sensitivity spanning multiple orders of magnitude. Mammals are estimated to have thousands of unique microRNAs ^39^, and panels of specific dysregulated miRs have been proposed as potential diagnostic markers for many disease states, including Alzheimer’s ^40^, infectious diseases ^41^ and a wide range of cancers ^42^. For DMD, three ‘dystromiRs’ (miR-1, miR-133a and miR-206) have been proposed as biomarkers ^43– 45^: these miRs are striated muscle-specific and are dramatically elevated in both DMD patients and the *mdx* mouse, and have been reported to fall in response to therapeutic exon skipping ^43^. These dystromiRs are also elevated in serum of canine models of DMD, such as the CXMDJ ^46^ and GRMD ^47^.

The aim of this study was to establish a panel of blood-borne musculoskeletal disease biomarkers that distinguish between the DE50-MD dog model and WT dogs, with the hypothesis that the DE50-MD dog biomarker profile reflects that of human DMD and further, that these biomarkers will be of sufficient sensitivity and stability to serve as outcome measures in future DE50-MD pre-clinical treatment trials.

## Methods

**Animal husbandry:** Dogs used in this study were from the DE50-MD colony, housed in a dedicated facility at the Royal Veterinary College (RVC), London. WT dogs were housed with DE50-MD dogs (according to temperament and hierarchy) in indoor kennels (12-hour light/dark cycle, 15–24 °C). Study dogs were typically housed in groups of 2/3. Kennel size varied slightly, but was approximately 4.5 m ^2 ^for a small kennel and 7.5 m ^2^ for a large kennel; the Animal (Scientific Procedures) Act 1986 (ASPA) code of practice (Section 2) regarding number of dogs per area of kennel size was followed at all times. Dogs had daily access (typically from 8 am to 3 pm) to large outdoor paddocks (approximately 100 m ^2^) in group sizes of up to 5 dogs: conditions that exceed the minimum stipulations of ASPA and according to RVC local Animal Welfare Ethical Review Body approval. Carrier female Beagle (RCC strain, Marshall Bioresources)-cross dogs, derived from an original founder Bichon-Frise cross Cavalier King Charles Spaniel female carrier, were mated with male Beagles (RCC strain) to produce all dogs for this study. Pregnant females (single housed) whelped naturally and puppies were kept with the mother in a large kennel with a heat lamp (~28 °C) to allow nursing. All puppies were microchipped at seven days of age. Puppies were reared by their mother until approximately four weeks, after which they were transitioned to puppy food (Burns) suitable to requirements: puppies under six months were fed at least three times daily with milk or tinned food and/or had ad-lib dry puppy food available. From six months onwards, dogs received two feeds daily and ad lib water. Dogs received daily human interaction and underwent a comprehensive socialisation programme. Dogs not required for studies were rehomed.

**Figure 1.**
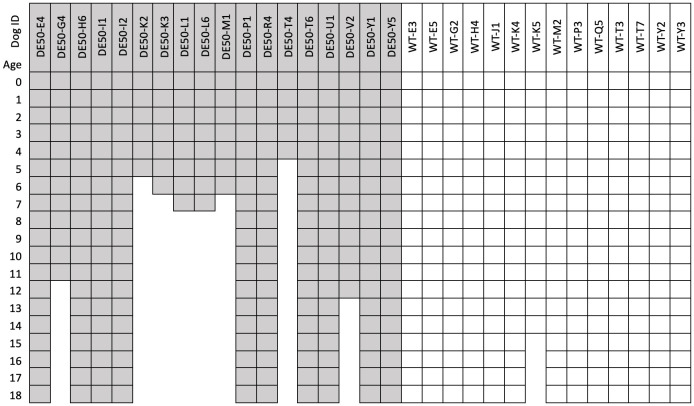
Blood sampling ages (in months) for dogs studied longitudinally. Dog ID shows the study name of each individual animal included in the longitudinal study: dog IDs with the preface “DE50-" (light grey) are DE50-MD dogs, dogs with the preface WT (white) are wild-type dogs.

**Study population:** All dogs used in the study were male. In total, data from 37 DE50-MD dogs and 35 WT dogs are included in this study (total 72); of these, 18 DE50-MD dogs and 14 WT dogs were studied longitudinally between 0 and 18 months of age ( Figure 1). Sample size for the longitudinal study was determined in order to generate an accurate estimation of the variance for each time point and genotype, to enable future sample size calculations, as reported in this paper. The remaining animals were male puppies, with data compiled from routine genotyping (perinatally) comprising Sanger sequencing of the DE50-MD mutation region and plasma CK activity measured within the first 8 days of life from a further 19 DE50-MD dogs and 21 WT dogs (see *Underlying data*); these dogs were subsequently, either rehomed (WT dogs only) or recruited to other studies. N values for each measurement are shown below each figure. Dependent on the biomarker analysed, it was not always possible to test samples from all timepoints for each dog. Missing data-points were a consequence of exhausting the finite supply of blood or tissue sample collected at a specific time-point, unless otherwise stated. Full details of samples tested for each biomarker can be found in *Underlying data*.

Genotyping was performed on cheek-swab derived DNA by PCR and Sanger sequencing as previously described ^8^. Briefly, cells were collected from the inside of the dog’s cheek using a bristled cheek swab. Genomic DNA was extracted from the cells, and the region of the *DMD* gene containing the DE50-MD mutation was amplified by polymerase chain reaction (PCR). PCR products were sequenced externally (Sanger sequencing by GATC biotech/Eurofins). With the exception of confirming the genotype as WT male or DE50-MD male, no additional inclusion or exclusion criteria were used when recruiting dogs to the study. Researchers involved in data acquisition and data analysis for this study were not blinded to genotype.

**ARRIVE guidelines:** ARRIVE guidelines were followed for the design and conduct of the study and an E10 checklist completed (see *Underlying data*). All experimental procedures involving animals in this study were conducted according to UK legislation, within a project licence (P9A1D1D6E, granted 11 June 2019) assigned under the Animal (Scientific Procedures) Act 1986 and approved by the Royal Veterinary College Animal Welfare Ethical Review Body (AWERB). All efforts were made to minimise any animal suffering throughout the study. Pre-determined end-points for DE50-MD dogs were established including dehydration (unresolved by fluid treatment), lethargy/motor dysfunction, weight loss/dysphagia, dyspnoea, listless behaviour/demeanour, or heart failure. Dogs were observed daily by animal technician staff and those showing any of these signs were reported to and assessed by the Study Director, the Named Veterinary Surgeon (NVS) and the Named Animal Care and Welfare Officer (NACWO). Should a dog reach any of the pre-determined end-points prior to the planned 18-month study end, they were humanely euthanised. Euthanasia was performed using an overdose of sodium pentobarbital (250 mg/kg) administered intravenously via preplaced catheter. Of the 18 DE50-MD dogs that were followed longitudinally, 11 were euthanised at the end of the planned 18-month study period (for research purposes unrelated to this study), 6 DE50-MD dogs were euthanised prior to 8 months of age as a result of reaching pre-determined humane end-points (related to dysphagia), 1 further DE50-MD dog (DE50-V2) was euthanised at 12 months of age due to dysphagia, and 1 DE50-MD dog (DE50-G4) was euthanised at 11 months of age due to developmental elbow dysplasia, believed unrelated to the DMD phenotype ( Figure 1). Four of the 14 WT dogs that were followed longitudinally were euthanised humanely at the end of the planned 18-month study period (WT-G2, WT-J1, WT-K4 and WT-M2) for research purposes unrelated to this study, and one further WT dog (WT-K5) was euthanised at 14-months of age due to developing steroid-responsive meningitis, a condition known to affect the Beagle breed ^48^. The remaining nine WT dogs were re-homed ( Figure 1).

### Blood sampling

Blood was obtained at approximately monthly intervals by jugular venepuncture into plain (serum) and lithium heparin (plasma) tubes. Blood samples were centrifuged (Heraeus, #3328) at 500 x g for 10 minutes at 4 °C. Serum or plasma (as appropriate) was aspirated, aliquoted and frozen at -80 °C until analysed.

### CK activity

CK activity was quantified in lithium heparinised plasma, using a Ilab600 (Instrumentation Laboratory) clinical chemistry analyser. 

### MYOM3 western blot

Serum samples were diluted 1:1000, and 5 μl of diluted sample was combined with 5 μl of loading buffer (4% sodium dodecyl sulphate (Applichem, #A0676), 20% glycerol (Sigma, #G5516), 0.004% bromophenol blue (Sigma, #B0126), 0.125 M Tris-HCl pH 6.8 (Sigma, #T3253), 10% β-mercaptoethanol (Sigma, #M7154)). Samples were then loaded into Tris/Glycine PAGE gels (7.5% Mini-PROTEAN TGX precast gels, Biorad, #4561026). All samples were tested in duplicate, on replicate gels. Gels were placed into an electrophoresis chamber (Biorad, #1658005EDU) with running buffer (10x TGS Buffer, Bio-Rad, #161-0732) and run at 90 volts, for 90 minutes. Proteins were transferred from the gel to a polyvinylidene difluoride blotting membrane (GE Healthcare, #10600023) by incubation in the electrophoresis chamber with transfer buffer (10x TG Buffer, Bio-Rad, #161-0734) at 300 amps for 90 minutes. Membranes were blocked in 10% milk powder (Marvel) in phosphate buffered saline (PBS, Fisher Scientific, #BR0014G)/Tween 0.05% (Sigma, #P9416) for one hour at room temperature, before incubation with a polyclonal rabbit anti-human MYOM3 antibody (Proteintech, #17692-1-AP, 1:1000 dilution) and a polyclonal rabbit anti-canine albumin antibody (as internal loading control, Biorbyt Ltd, #orb242465; 1:1,000,000 dilution) overnight at 4 °C. The anti-human MYOM3 antibody has confirmed cross-reactivity with canine MYOM3 ^24^. The following day, membranes were washed over an hour in PBS/Tween 0.05% before incubation with a horseradish peroxidase-conjugated polyclonal goat anti-rabbit IgG secondary antibody (Dako, #P0448, 1:10,000 dilution, one hour at room temperature). Membranes were developed using Enhanced chemiluminescence (ECL - ThermoFisher, #32106), and were imaged on a ChemiDoc ^TM^MP Imaging System (Bio-Rad). After imaging, signals were quantified via densitometry using ImageJ software. Probing for MYOM3 gives 2 clear bands at approximately 100 kDa and 140 kDa (as seen in previous studies ^24^), while albumin is detected at approximately 55 kDa. Densitometry data for both MYOM3 bands was combined and normalised to albumin. 

### MSTN ELISA

MSTN was quantified in serum samples by sandwich enzyme-linked immunosorbent assay (ELISA) according to the manufacturer’s instructions (R&D Systems, #DGDF80). Samples were first activated by incubation of 20 μL of test serum with 10 μL 1 N HCl for 10 minutes at room temperature, followed by addition of 10 μL 1.2 N NaOH/0.5 M HEPES. Samples were then diluted to a final dilution of 1:20 by addition of 360 μL of calibrator diluent (supplied with ELISA kit). All samples were tested in duplicate.

### DystromiR RT-qPCR

**RNA isolation and cDNA synthesis:** microRNAs were isolated from frozen serum samples (100-200 μl per sample point) using the mRNeasy serum/plasma kit (Qiagen, #217184). cDNA was prepared via miScript II RT kit (Qiagen, #218161) using 3 μl of serum RNA (low RNA content of serum precludes accurate quantification via nanodrop). All cDNA preparations were subsequently diluted 1:20 with nuclease free water.

**Quantitative PCR (qPCR):** Following cDNA synthesis, qPCR was conducted via the miScript PCR System (Qiagen, #218073) in 10 μl vols (2 μl cDNA) using a CFX384 light-cycler (BioRad), with primers specific to *Canis familiaris* miR-1 (Qiagen, #MS00029337), miR-133a (#MS00029498) and miR-206 (#MS00030009) along with a universal primer (Qiagen, #218073). A melt curve was included in each run. Pilot work suggested miR-23a (primers: Qiagen, #MS00030170) and miR-223 (#MS00030126) were suitable references, and these two miRs were robustly expressed in all samples (see *Underlying data*). Analysis of our full dataset however revealed a modest but significant increase in miR-23a within dystrophic samples. All samples were thus normalised to miR-223 alone (this miR exhibited no significant disease association). As a further QC step, any samples with miR-23a/miR-223 Cq values nearing the stochastic range (Cq>27, indicating low RNA recovery or poor cDNA synthesis) were excluded from analysis: 8 samples (out of 207) were rejected in this manner (6 WT, 2 DE50-MD).

### Statistical analysis

Linear mixed models were used to determine the effects of age, genotype and their interaction for CK activity, MYOM3, MSTN, and miRs. *Post hoc* analysis was performed using Tukey’s multiple comparisons test. A principal component analysis was used to reduce dimensions of the full biomarker dataset (CK activity, MYOM3, MSTN, and miR-1, -133a and -206). P values of less than 0.05 were considered statistically significant. Linear mixed modelling, *post hoc* analyses and the principal component analysis were performed using IBM SPSS Statistics Version 28 and graphs were produced using Graphpad Prism 8.0. Free software alternatives could also be used, such as R or JASP. Estimates of sample size that would be appropriate for prospective therapeutic trials with the colony were performed using GLIMMPSE online software ^49^, using a repeated measures model and a desired power of 0.8. Data was tested for normality by Shapiro-Wilk test and, where appropriate, data was log transformed prior to statistical analysis. Comparison of biomarker data from DE50-MD dogs that were euthanised prior to 8 months of age with age-matched DE50-MD dogs that were maintained for the full 18-month study period revealed no statistical differences for any marker evaluated (linear mixed model adjusted for repeated measures, P>0.05, see *Extended data*), thus all data from these six animals was included in our analysis.

## Results

### CK activity

**Figure 2.**
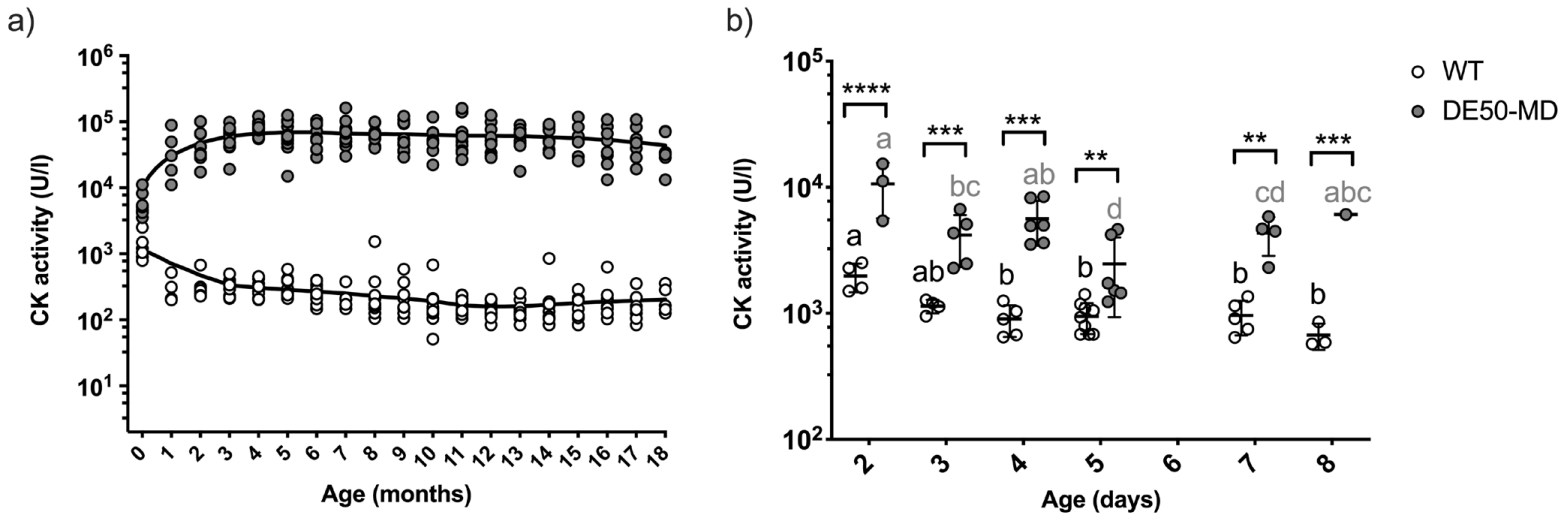
Plasma CK activity in DE50-MD and WT dogs. Figure **a**) data points represent individual DE50-MD (grey) or WT (white) dogs at different ages. Dogs were studied longitudinally between 1 and 18 months of age. DE50-MD: total N=18 dogs, N=5-17 per age group; WT: total N=14 dogs, N=4-14 per age group. Data points at age 0-months represent samples taken within the first week of life. Lowess curves show the general trend in CK activity with age in each genotype. Plasma CK activity was significantly higher in DE50-MD compared to WT dogs at all timepoints (linear mixed model analysis adjusted for repeated measures, P<0.0001). Figure **b**) CK activity in DE50-MD (grey, N=28 dogs) and WT (white, N=26 dogs) plasma samples collected within the first 8 days of life. Asterisks denote the level of significance of a difference between genotypes based on linear mixed model analysis: ** P<0.01, *** P<0.001, **** P<0.0001. (CK: creatine kinase; WT: wild type.)

Monthly measurement of plasma CK activity revealed substantially higher levels in DE50-MD dogs than WT dogs at all timepoints (P<0.0001; Figure 2) ^50^. There was considerable variation in CK activity within DE50-MD dogs (mean = 60600 U/l +/- 30200 standard deviation (SD); range: 3500-160000 U/l; N=18), but levels were consistently elevated above those found in WT dogs (WT range: 50-2500 U/l; N=14). There was a modest, but significant, interaction between CK activity and age in both genotypes (DE50-MD: P=0.003; WT: P<0.0001), however the trend of this effect was different between the two genotypes (P=0.003). A fitted Lowess regression curve showed that DE50-MD plasma CK activity steadily increased up to 5 months of age, with a subsequent gradual fall from five to 18 months ( Figure 2a). In contrast, WT plasma CK activity decreased from one to 10 months of age, at which point it plateaued until the final time-point of 18 months. Differences in plasma CK activity were detectable at very young ages: CK activity in animals aged two to eight days old was significantly higher in DE50-MD dogs compared to WT (P<0.01, Figure 2b).

### Myomesin 3 (MYOM3)

**Figure 3.**
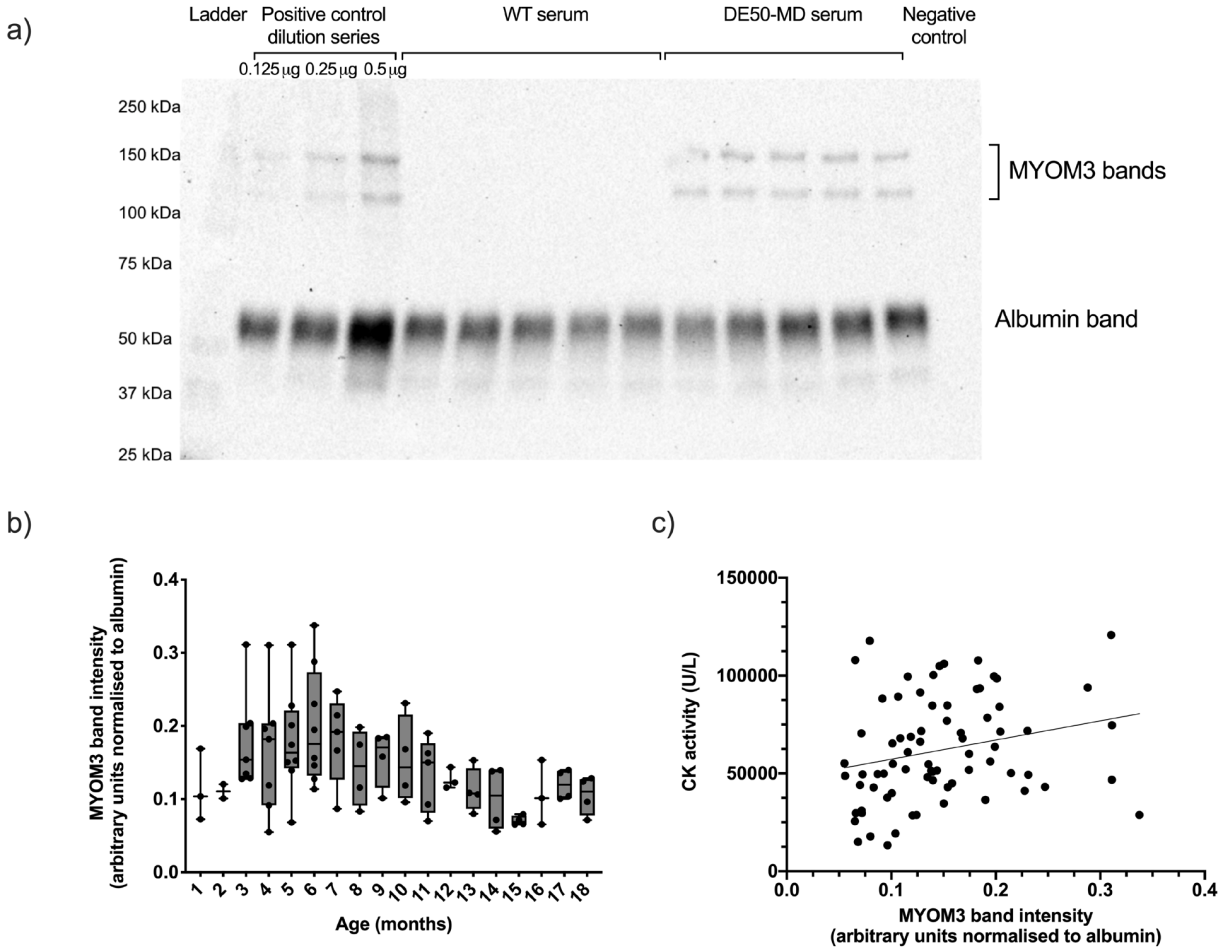
MYOM3 in serum samples from DE50-MD and WT dogs. Figure **a**) representative MYOM3 western blot. This western blot shows a positive control mini-dilution series that was run alongside samples in every gel, followed by 5 samples of WT control dog serum, 5 samples of DE50-MD dog serum, and a negative control lane in which no protein was loaded. Figure **b**) MYOM3 quantified by western blot in serum samples from dogs studied longitudinally between 1 and 18 months of age. DE50-MD: grey, total N=83 samples, from N=10 dogs, N=2-8 per age group; WT dogs: (not shown, see *Underlying data*), total N=113 samples, from N=10 dogs, N=3-9 per age group. Bands corresponding to MYOM3 were not detected in any WT serum samples tested. Boxes extend from the 25 ^th^ to 75 ^th^ percentile, with a line within the box at the median value. Each point represents an individual sample, and whiskers show the minimum and maximum results for that age-group. Band intensity calculated by densitometry using ImageJ software. Figure **c**) correlation between MYOM3 quantity and CK activity in blood samples from DE50-MD dogs studied longitudinally between 1 and 18 months, total of N=75 samples, from N=10 dogs. (MYOM3: myomesin-3; WT: wild type.)

Serum MYOM3 protein was significantly associated with genotype (P<0.0001), being robustly detected in all DE50-MD serum samples tested (total DE50-MD samples N=83, from 10 different dogs, N=2-8 per age group, Figure 3) ^51^, but not found in any WT sample tested (total WT samples N=113, from 10 different dogs, N=3-9 per age group). Levels of MYOM3 in DE50-MD serum ranged from 0.06-0.34 (mean 0.15 +/- 0.06, arbitrary units: albumin-normalised band intensity), and exhibited a significant effect of age (P<0.001): MYOM3 levels peaked at 6 months and declined thereafter. Between three and five months of age, one particular DE50-MD dog (DE50-L1) had substantially higher MYOM3 levels than any other age-matched dog ( Figure 3b). This dog was euthanised at seven months of age due to dysphagia (a pre-defined humane end-point of the study). Serum MYOM3 in DE50-MD dogs positively correlated with plasma CK activity (linear mixed model estimate of slope gradient: 101000 +/- 30000 SE, P=0.001, Figure 3c). 

### Myostatin (MSTN)

**Figure 4.**
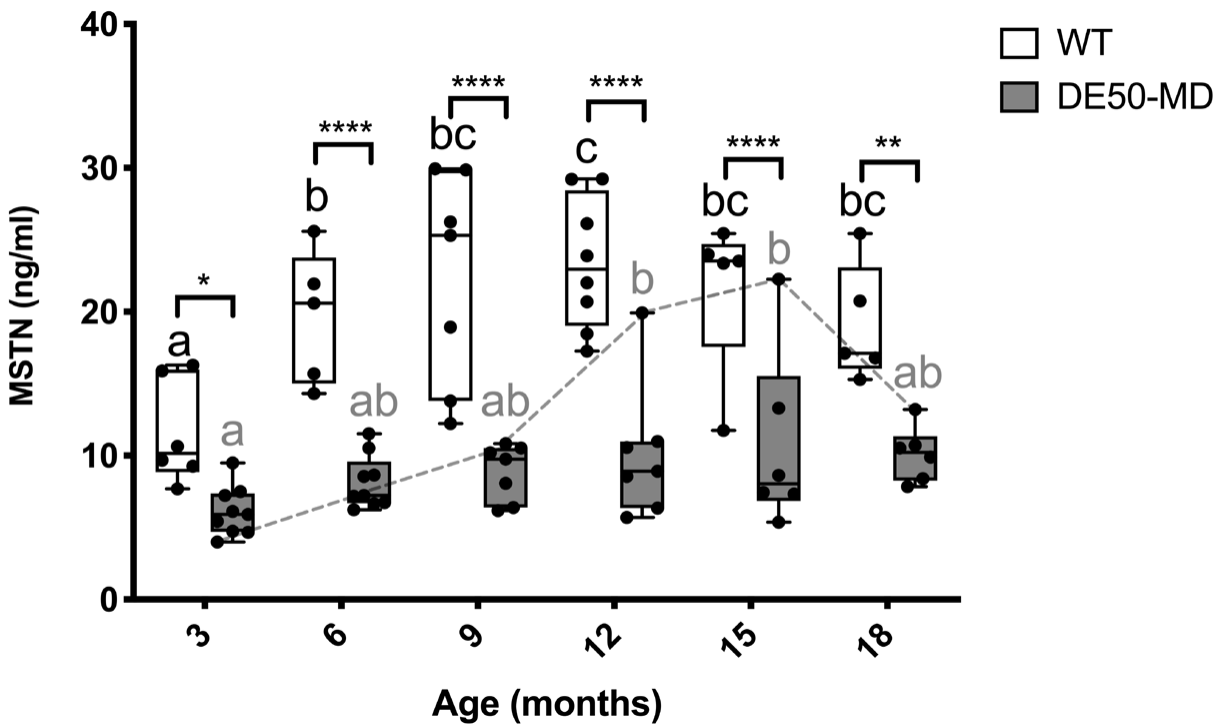
MSTN concentration in serum samples from DE50-MD and WT dogs. Samples were collected at 3-monthly intervals (from 3 to 18 months of age) from DE50-MD and wild-type (WT) control dogs studied longitudinally. DE50-MD: grey, total N=10 dogs, N=6-9 per age group; WT: white, N=8 dogs, N=5-8 per age group. Boxes extend from the 25 ^th^ to 75 ^th^ percentile, with a line within the box at the median value. Each point represents an individual sample, and whiskers show the minimum and maximum results for that age-group. Asterisks denote the level of significance of a difference between genotypes based on linear mixed model analysis adjusted for repeated measures: *P<0.05, **P<0.01, ****P<0.0001. Letters a, b and c denote statistically significant differences (P<0.05) in the mean within either the DE50-MD (grey letters) or WT (black letters) genotypes: means sharing a letter are not significantly different within each genotype group. (MSTN: myostatin; WT: wild type.)

Serum MSTN concentration was, on average, 2.3-fold higher in WT serum compared to DE50-MD serum across all ages measured (P<0.05; DE50-MD: mean MSTN = 8.8 ng/ml +/- 3.5 SD; WT: mean MSTN 19.8 ng/ml +/- 6.3 SD; Figure 4). In the WT cohort, MSTN concentration peaked at nine months of age, remaining at this level for all subsequent age points. Within the DE50-MD genotype, mean MSTN concentration peaked at a later stage (between 12–15 months), though closer examination reveals one DE50-MD dog exhibited markedly higher MSTN concentration than all other age-matched DE50-MD dogs between 12 and 18 months (DE50-I2, Figure 4 dashed grey line). Excluding this individual from analysis removed any age-associated differences in MSTN within the DE50-MD genotype.

### DystromiRs

**Figure 5.**
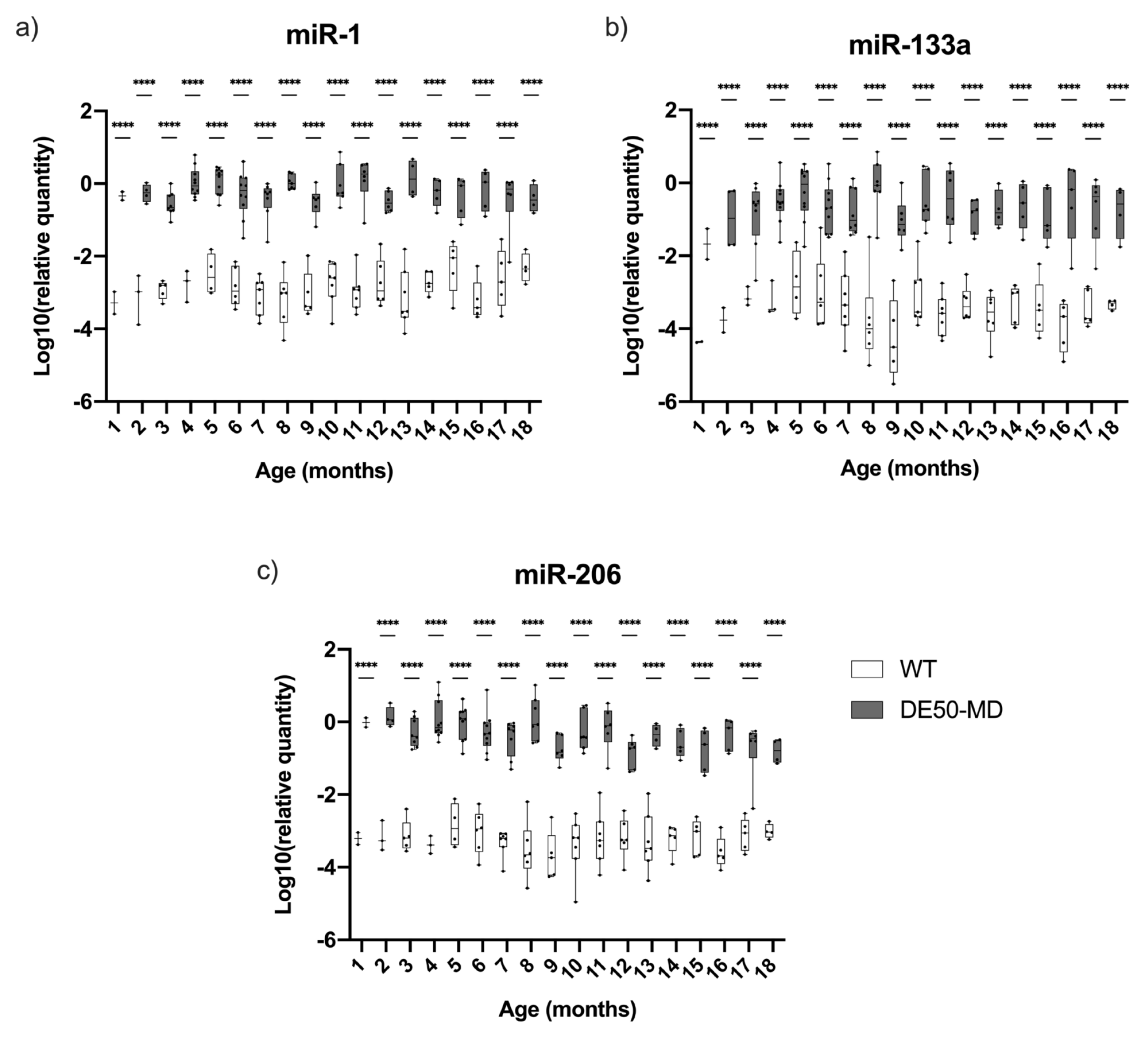
Relative dystromiR quantity in serum samples from DE50-MD and WT dogs. Figure **a**) miR-1, **b**) miR-133a, the grey dashed trace indicates miR-133a results for a single healthy dog (WT-H4) that rose to near-DE50-MD levels between 5 and 8 months of age, and **c**) miR-206. DystromiR concentrations were normalised to a reference miR: miR-223 (see methods). Samples were taken from DE50-MD and wild-type (WT) control dogs studied longitudinally between 1 and 18 months of age. DE50-MD: grey, total N=12 dogs, N=2-10 per age group; WT: white, N=8 dogs, N=2-8 per age group. Boxes extend from the 25 ^th^ to 75 ^th^ percentile, with a line within the box at the median value. Each point represents an individual sample, and whiskers show the minimum and maximum results for that age-group. Asterisks denote the level of significance of a difference between genotypes based on linear mixed model analysis adjusted for repeated measures: ****P<0.0001. (WT: wild type.)

All three dystromiRs were markedly elevated in DE50-MD serum ( Figure 5): under linear mixed model analysis, genotype was highly significant (P<0.0001) for all three miRs, while the effect of time was not significant for any (though a modest interaction of genotype and time was detected). All three miRs were significantly elevated at all time points (P<0.0001, Figure 5). Although the mean quantity for each of the three miRs was markedly higher in DE50-MD compared to WT samples, there was considerable variation within age groups for both genotypes ( Figure 5).

Raw Cq data indicated that serum concentrations of these three miRs were typically very low in healthy samples (often nearing the lower limit of detection), while levels in DE50-MD serum were more robust (3–4 orders of magnitude greater, comparable with levels of the reference, miR-223). This implies that these miRs are not typically present in healthy serum. In one instance, concentration of serum miR-133a rose to near-DE50-MD levels in a single healthy dog (WT-H4, indicated by the grey dashed trace, Figure 5b). This specific miR remained elevated for four consecutive months (months 5–8), while levels of miR-1 and miR-206 remained comparable with other healthy animals. We were unable to determine the cause of this increase, but this finding highlights the importance of adequately-powered sample cohorts and longitudinal data collection.

**Figure 6.**
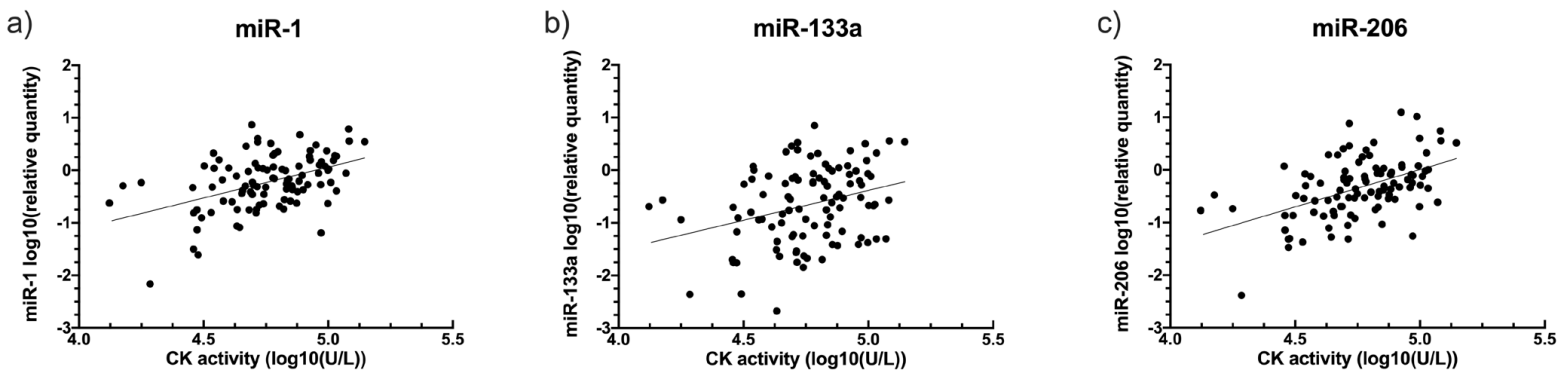
Relationship between creatine kinase (CK) activity and relative quantity of dystromiRs in DE50-MD dog blood samples. Figure **a**) miRs-1 **b**) miR-133a and **c**) miR- 206. Linear regression calculated based on linear mixed model analysis, accounting for repeated measures. Samples were taken from DE50-MD and wild-type (WT) control dogs studied longitudinally between 1 and 18 months of age. Each point represents an individual sample (N=101 for each miR) from a total of N=12 DE50-MD dogs. No relationship was found between CK activity and the relative quantity of any of the miRs in WT samples (not shown, see *Underlying data*) (P>0.05). (CK: creatine kinase; WT: wild type.)

CK activity positively correlated with relative quantity of each of the three miRs in DE50-MD samples (miR-1: slope gradient estimate: 1.1 +/- 0.3 SE, P<0.00001; miR-133a: slope gradient estimate: 0.9 +/- 0.3 SE, P=0.003; miR-206: slope gradient estimate: 1.3 +/- 0.2 SE, P<0.00001; Figure 6), but not in WT samples (miR-1: P=0.53; miR-133a: P=0.27; miR-206: P=0.73; see *Underlying data*).

### Principal component analysis

**Figure 7.**
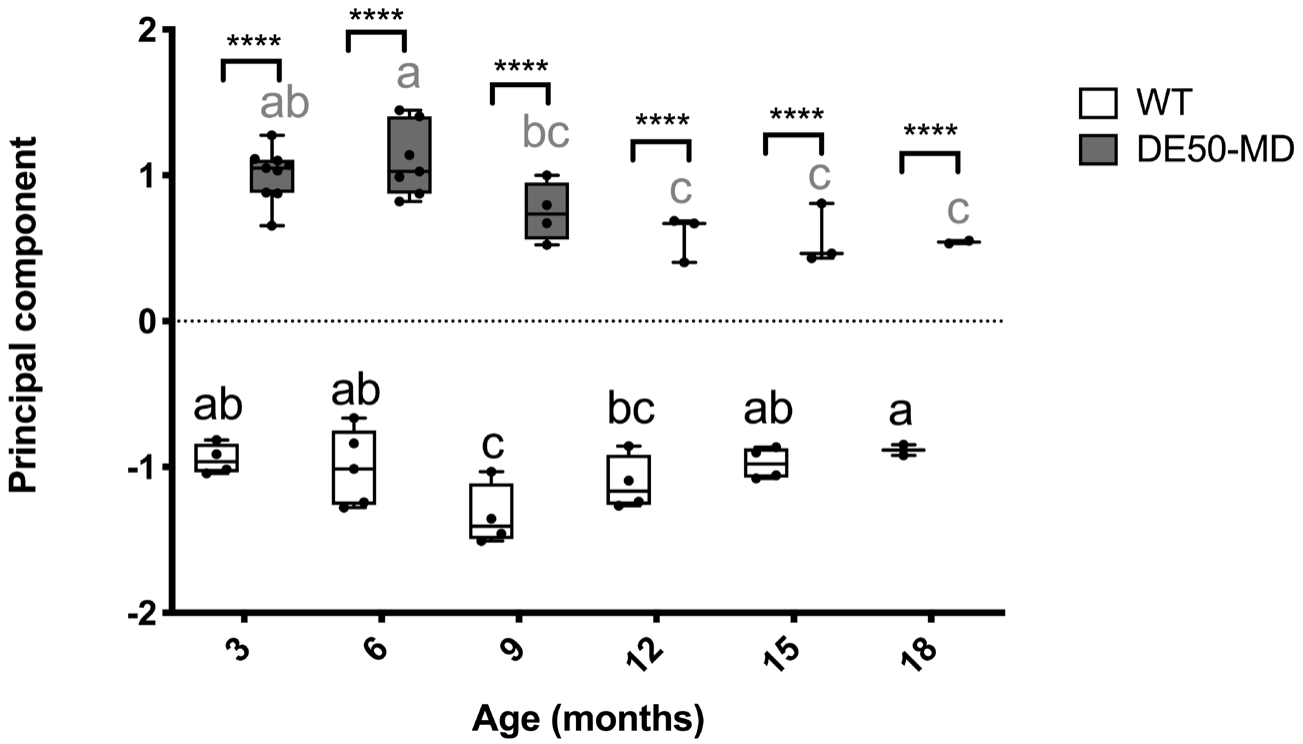
Principal component analysis of serum and plasma biomarkers. Included in the analysis was plasma CK activity (log10 U/l), serum MYOM3 quantity (AU), serum MSTN concentration (ng/ml), serum miR-1, miR-133a and miR 206 levels (log10 relative quantity). Study population consisted of DE50-MD and wild-type (WT) dogs studied longitudinally between 3 and 18 months of age. DE50-MD: grey, N=10 dogs total, N=2-9 per age group; WT: white, N=7 dogs total, N=2-5 per age group. Where an individual was missing data for a specific biomarker, missing data was replaced with the mean of the relevant genotype and age-group for that biomarker. Boxes extend from the 25 ^th^ to 75 ^th^ percentile, with a line within the box at the median value. Each point represents an individual sample, and whiskers show the minimum and maximum results for that age-group. Asterisks denote the level of significance of a difference between genotypes based on linear mixed model analysis: ****P<0.0001. Letters a, b and c denote statistically significant differences (P<0.05) in the mean within either the DE50-MD (grey letters) or WT (black letters) genotypes: means sharing a letter are not significantly different within each genotype group. (CK: creatine kinase; WT: wild type.)

A principal component analysis was performed to evaluate all six musculoskeletal blood-borne biomarkers quantified in this study. Dogs that were missing data for more than one biomarker were excluded from analysis; when a single biomarker was missing for an animal, the missing value was replaced with the mean within the relevant genotype and age-group (for raw data see *Underlying data*). One principal component was extracted that explained 84% of the variation within the dataset. Values for the principal component were substantially higher for DE50-MD dogs than WT dogs at all ages (three to 18 months; Figure 7). There was a peak in the principal component score at six months in the DE50-MD dogs, and a subsequent decrease to a plateau between months 12 to 18. In contrast, WT dogs exhibited a decrease in the principal component score between months three to nine, followed by a gradual increase through to month 18.

### Sample size calculations for future trials

Sample sizes that would likely be appropriate for future prospective trials of therapeutics were calculated for all biomarkers identified in the study, and for the derived principal component identified above. Principal component output (AU), CK activity (log10 U/L), MYOM3 (AU), MSTN (ng/ml), and miR-1, -133a and -206 (log10 relative quantities) all required very small numbers of dogs (seven or fewer) per genotype to detect as little as a 25% improvement in the biomarker result towards WT levels ( Table 1). Note that the large inter-individual variation and skew within CK activity and miR quantity datasets necessitated log transformation for statistical analyses, therefore percentage improvements are based on log values for this subset of biomarkers.

**Table 1.** Sample size calculations. N required to show with sufficient power (0.8) an improvement in a DE50-MD biomarker towards WT levels with any given treatment. Sample sizes were calculated for the principal component analysis output (performed on blood-borne CK activity, MYOM3 quantity, MSTN concentration, and miR-1, -133a and -206 relative quantity) and for each individual biomarker that was significantly elevated in DE50-MD compared to WT blood samples for dogs aged 3–18 months. (CK: creatine kinase; MSTN: myostatin; MYOM3: myomesin-3; WT: wild type.)

	Percentage improvement towards WT concentrations			
	(N per genotype)			
Biomarker	25%	50%	75%	100%
Principal component	3	2	2	2
CK activity (Log10 U/l)	3	2	2	2
MYOM3 (AU)	6	3	3	2
MSTN (ng/ml)	7	3	3	2
miR-1 (Log10 relative quantity)	5	3	2	2
miR-133a (Log10 relative quantity)	7	3	3	2
miR-206 (Log10 relative quantity)	4	2	2	2

## Discussion

The purpose of this study was to evaluate candidate blood biomarkers of musculoskeletal disease in the DE50-MD dog model of DMD, for use in future pre-clinical longitudinal trials. Our focus was to identify biomarkers demonstrating sufficient consistency and sensitivity to allow detection of even modest amelioration of disease (such as might be found in early responses to therapeutic intervention). Given the minimally-invasive nature of blood sampling, a panel of such biomarkers could then be used to monitor disease progression in extended therapeutic trials in DE50-MD dogs.

Plasma creatine kinase (CK) activity in the DE50-MD dog model was markedly elevated at all ages and showed a similar trend to that seen in human DMD patients ^52^, peaking earlier in life and then subsequently decreasing. As release from damaged muscle tissue represents the principal source of circulatory CK, the slow decline with age observed in DE50-MD dogs likely reflects both systemic decreases in muscle mass, and reduced exercise frequency/intensity as disease progresses. In the DE50-MD dog, muscle volume increases until about nine months of age, at which point it plateaus through to at least 18 months of age. However, MRI results suggest that an increasing proportion of normal muscle tissue is replaced by fibrotic tissue and/or other cell infiltrates as the disease progresses ^53^. In addition, preliminary activity monitoring data suggests that DE50-MD dog activity steadily decreases between three and 14 months of age, at which point activity intensity plateaus at a low level through to 18 months of age (Karimjee, Piercy *et al*., unpublished work). These findings support the theory that a decrease in normal muscle tissue mass and activity intensity could be contributing to the gradual decline in CK activity in DE50-MD serum with age.

As seen in other animal models of DMD (and in human patients), plasma CK activities varied considerably between individual animals in the DE50-MD colony ^52, 54, 55^. Such variation reflects the marked sensitivity of this metric and hence its possible drawbacks when using it to demonstrate low to moderate therapeutic efficacy ^21, 56^. A substantial decline in CK activity would be required to demonstrate a treatment effect, but given its widespread clinical use and the low proposed sample sizes calculated in this study, we recommend its continued use. 

MYOM3 was investigated as an alternative or adjunct DMD musculoskeletal biomarker, as this molecule exhibits lower inter-individual variation than CK, and is less influenced by age ^24^. The presence of MYOM3 fragments in serum was an excellent biomarker for differentiating between the DE50-MD and WT dog genotypes, and was markedly less variable between dogs than CK activity. A study in the GRMD model by Rouillon *et al.* ^24^ suggested that serum MYOM3 in the GRMD model might be age-independent up to at least 18 months of age, based on the finding that MYOM3 expression was very similar in two and 18-month-old dogs. In our DE50-MD model, continuous data from monthly blood samples (between one and 18 months) revealed that serum MYOM3 increased gradually from one month of age to a peak at six to seven months and gradually decreased thereafter. This finding correlates with the trend seen in CK activity over the 18 month DE50-MD study period, and is supported by the fact that levels of MYOM3 in human DMD patient samples are also affected by age, with patients aged three to 10 years exhibiting concentrations five times higher on average than patients aged 12 to 20 years ^24^. On a per-sample basis, however, we found only very weak correlation between CK and MYOM3 in the DE50-MD dogs; this might reflect the labile nature of CK release in damaged muscle, perhaps related to recent exercise ^55^ and the fact that elevations in serum/plasma CK activity might result from relatively minor loss of sarcolemmal integrity, whereas increases in MYOM3 might reflect more substantial muscle degeneration.

Another circulating biomarker that reliably distinguished between the DE50-MD and WT genotype is the muscle growth inhibitor MSTN. Serum MSTN was lower in DE50-MD compared to WT at all ages tested (three-monthly intervals, from three to 18 months of age), which agrees with recent data from the GRMD dog model ^38^. Unlike CK and MYOM3, DE50-MD serum MSTN concentration did not decline with age, though as this biomarker is found at reduced concentration in DE50-MD serum (rather than the elevated CK and MYOM3), further reductions might be more challenging to detect. As MSTN is produced by skeletal muscle, decreased serum MSTN concentration might primarily be a consequence of lower overall muscle mass in DE50-MD dogs as compared to age-matched WT controls ^53^. However, recent gene expression analysis of DMD patient and GRMD muscle biopsy samples suggests that there is active downregulation of the MSTN pathway ^35, 38^. Further work to evaluate the MSTN pathway in DE50-MD muscle and the influence of relative muscle mass, could help elucidate the mechanisms behind reduced MSTN in dystrophic individuals. Nonetheless, based on our sample size calculations, serum MSTN is an excellent biomarker of the DE50-MD phenotype.

Serum microRNAs (miRs) represent further biomarker candidates: use of qPCR to measure these molecules results in very high sensitivity, allowing accurate quantification over multiple orders of magnitude. We examined the presence of the three dystromiRs: miR-1, miR-133a and miR-206 in both healthy and DE50-MD serum, with levels in the latter being several orders of magnitude greater than in the former at all time points measured, suggesting that these three microRNAs are strong candidate biomarkers. The levels of these miRs in dystrophic serum are comparable with other, non-disease associated serum miRs (such as the reference miR-223), but their levels in healthy serum are close to zero. Serum miR-133a (alone) was however detected at elevated levels in one WT dog for four consecutive months, a finding which suggests additional factors might influence serum miR content, but also illustrates the advantages of assessing multiple microRNAs. Indeed, the dystromiRs are a subset of a larger group of microRNAs known as the myomiRs (which also include miR-208, miR-486 and miR-499): these miRs are enriched in healthy skeletal muscle tissue, and their expression is held to be associated with myoblast differentiation, fibre-type choice and muscle regeneration ^57^. Tissue myomiR concentrations alter in response to exercise and loading, however the presence of these microRNAs within serum is typically only found following bouts of extreme exercise (such as in marathon runners), with levels declining within days ^58^. This indicates that, like CK and MYOM3, these markers are usually restricted to muscle, with their entering the circulation only as a consequence of damage rather than physiological release. Serum dystromiR levels might consequently be expected to decline not only following therapeutic intervention, but also with age- and disease-related muscle wasting (as with serum CK ^59, 60^ and MYOM3 ^24^, above). Our analysis did reveal a modest but significant effect of genotype and age combined, but not of age alone: this implies that miRs do not globally alter with age, but might nevertheless do so in a genotype-specific fashion. Supporting this, miR-206 exhibited a modest decline with increasing age in dystrophic serum. Nevertheless, even in the oldest DE50-MD dogs (18 months), levels of all three miRs remained markedly elevated over healthy values, thus any muscle wasting-associated decline in their serum concentration is mostly likely modest in this model. DystromiRs consequently appear to represent a biomarker with comparable sensitivity and dynamic range to CK activity, though possibly with some of the same caveats attributed to CK; future work to assess dystromiR quantity following a bout of exercise or a therapeutic intervention will further determine their lability and utility in pre-clinical trials using the DE50-MD model.

A principal component analysis (PCA) was performed to summarise the variation in the dataset of all biomarkers identified in this study. A single principal component was extracted that explained 84% of the variation in the dataset, indicating that the six biomarkers were highly correlated. This principal component distinguished the two genotypes for all age-groups tested, with sufficient power to detect a 25% improvement in DE50-MD results towards WT values with small N numbers according to our sample size projections. We propose that this combined approach might have advantages over use of individual biomarkers, as the assessment might be less influenced by intra-animal variation, extreme biomarker sensitivity and lability, and effects of exercise. 

When analysed individually, sample size calculations predict that CK activity, MYOM3, MSTN and relative quantities of miRs -1, -133a and -206 would require N numbers of seven or fewer dogs per genotype to detect at least a 25% improvement in DE50-MD values towards WT levels in response to interventions. To date, these blood biomarkers have not been evaluated in a longitudinal pre-clinical trial, thus whether they will normalise in response to a treatment, and the dynamics of such a change, is yet to be determined. One of the strengths of the DE50-MD natural history study, of which blood biomarker analysis comprises just one facet, is that we have identified a wide range of biomarkers (including the use of MRI ^53^, activity metrics, muscle physiology, cardiac muscle evaluation, neurological examination, and muscle histology (Piercy *et al.*, unpublished work)), maximising the likelihood of detecting any ameliorative response to therapy. By collating all of the results of the natural history study, we can see if the musculoskeletal blood-borne biomarkers identified in the current work correlate with other aspects of the DE50-MD phenotype. While we did not find any difference in circulating biomarker results between dogs that remained in the study for the full 18 months and dogs that were euthanised prior to 8 months for reasons relating to dysphagia, it is possible that the biomarkers might reflect other measures of clinical disease. Thus, future work will determine whether these blood-borne biomarkers can be used as indirect measures of severity for specific aspects of the DE50-MD phenotype.

Overall, our data suggests that this circulating musculoskeletal biomarker profile of the DE50-MD dog closely mimics that of human DMD patients, supporting the validity of the use of these dogs as an animal model for DMD. Of our candidate biomarkers, MYOM3 represents a valid alternative to CK activity as a marker of muscle structural damage, with the benefits of lower inter-individual variation. Serum MSTN concentration also reliably differentiates between genotypes, with the advantage of minimal age-associated variability. Lastly, our dystromiR panel represent high-sensitivity biomarkers that are present in DE50-MD serum at levels several orders of magnitude higher than WT dog serum. Analysing these biomarkers in combination also shows promise for future evaluation of therapeutics. This work contributes to establishing a repertoire of potential biomarkers in the DE50-MD dog. Future work will hence show if treatments that alleviate the dystrophic phenotype will restore these biomarkers towards WT dog levels, thus testing their efficacy for pre-clinical studies conducted in the DE50-MD dog model.

## Data availability

### Underlying data

Figshare: Underlying data - DE50-MD blood biomarker - Riddell, Hildyard, Harron, Wells, Piercy, https://doi.org/10.6084/m9.figshare.17032370 ^50^.

This project contains the following underlying data:

○ CK raw data.zip
○ MYOM3 raw data.xlsx
○ MSTN raw data.zip
○ MiR raw data.zip
○ PCA raw data.xlsx
○ Sample ID details for all biomarkers.docx

Figshare: Underlying data - DE50-MD blood biomarker – MYOM3 WB TIFFs - Riddell, Hildyard, Harron, Wells, Piercy, https://doi.org/10.6084/m9.figshare.17032430 ^51^.

This project contains the following underlying data:

○ MYOM3 WB TIFFs (Folder contains all raw MYOM3 western blot images)

### Extended data

Figshare: Extended data - DE50-MD blood biomarker - Riddell, Hildyard, Harron, Wells, Piercy, https://doi.org/10.6084/m9.figshare.17032436.

This project contains the following extended data:

○ Early vs late euthanasia raw data.xlsx
○ Early vs late euthanasia figures.docx

### Reporting guidelines

Figshare: ARRIVE E10 Checklist - DE50-MD blood biomarker - Riddell, HIldyard, Harron, Wells, Piercy.pdf, https://doi.org/10.6084/m9.figshare.17032370 ^50^.

Data are available under the terms of the Creative Commons Attribution 4.0 International license (CC-BY 4.0).
