# Supplementary material for: Longitudinal assessment of blood-borne musculoskeletal disease biomarkers in the DE50-MD dog model of Duchenne muscular dystrophy
Source: Wellcome Open Res. 2022 Aug 17;6:354. Originally published 2021 Dec 20. [Version 2] doi: 10.12688/wellcomeopenres.17398.2 (PMC9111359; doi:10.12688/wellcomeopenres.17398.2)
Supplement: Peer review history

# Peer review: Reviewer response for version 2

**DOI:** 10.21956/wellcomeopenres.20119.r52062

**Published:** 2022-08-18

**Authors:** Clare Rusbridge

**Affiliations:** 1 The School of Veterinary Medicine, University of Surrey, Guildford, UK; 2 Wear Referrals, Linnaeus Group, Durham, UK

The authors have answers my comments and queries satisfactorily.

Is the work clearly and accurately presented and does it cite the current literature?

Yes

If applicable, is the statistical analysis and its interpretation appropriate?

Yes

Are all the source data underlying the results available to ensure full reproducibility?

Yes

Is the study design appropriate and is the work technically sound?

Yes

Are the conclusions drawn adequately supported by the results?

Yes

Are sufficient details of methods and analysis provided to allow replication by others?

Yes

Reviewer Expertise:

I am a veterinary neurologist and a clinician that specialises in muscle disease.

I confirm that I have read this submission and believe that I have an appropriate level of expertise to confirm that it is of an acceptable scientific standard.

# Peer review: Reviewer response for version 2

**DOI:** 10.21956/wellcomeopenres.20119.r52061

**Published:** 2022-08-18

**Authors:** Laurent Tiret

**Affiliations:** 1 U955 - IMRB :: Inserm, Université Paris-Est Créteil, École nationale vétérinaire d'Alfort, Maisons-Alfort, France

In this version 2, the authors have accurately incorporated all the answers to my requests for clarification.

Is the work clearly and accurately presented and does it cite the current literature?

Yes

If applicable, is the statistical analysis and its interpretation appropriate?

Yes

Are all the source data underlying the results available to ensure full reproducibility?

Yes

Is the study design appropriate and is the work technically sound?

Yes

Are the conclusions drawn adequately supported by the results?

Yes

Are sufficient details of methods and analysis provided to allow replication by others?

Yes

Reviewer Expertise:

Comparative medical genetics, translational myology, mouse, rat & dog models of neuromuscular disorders

I confirm that I have read this submission and believe that I have an appropriate level of expertise to confirm that it is of an acceptable scientific standard.

# Peer review: Reviewer response for version 1

**DOI:** 10.21956/wellcomeopenres.19235.r50263

**Published:** 2022-05-16

**Authors:** Laurent Tiret

**Affiliations:** 1 U955 - IMRB :: Inserm, Université Paris-Est Créteil, École nationale vétérinaire d'Alfort, Maisons-Alfort, France

**Contextualized summary of the work**

 The data described here are part of a series of recently published papers on the precise longitudinal characterization of a cohort of dogs that are models of dystrophinopathy. Since the initial identification in 2010 of the loss-of-function mutation in the *DMD* gene (deletion of exon 50) in one of the two hotspot regions of the mutations catalogued in children, the investigators of the present study have carried out an exemplary work in a short period of time, allowing to gradually position this model as one of the most relevant for the preclinical validation of innovative therapeutic strategies at the individual level (precision medicine). 

 The biological question addressed here concerns the validation of blood biomarkers reflecting skeletal myonecrosis that would be the most discriminant within the group of affected dogs. A set of 6 blood-borne biomarkers (CK, MYOM3, MSTN, miR1, miR-133A and miR-206) was validated to serve as outcome parameters, individually (MYOM3 displaying less inter-individual variability than CK, MSTN showing less age-associated variability) or in combination with each other to confirm or invalidate the success of therapeutic strategies assessed on these dogs - 2 to 7 dogs per genotype would provide sufficient statistical power to conclude.

**Opinion**

 The article is very well written. The introduction provides a very well organized review of many key articles in the field.

 The data analysis was conducted rigorously. A principal component explaining 84% of the variation in the dataset including WT and DMD dogs was extracted and is sufficient to detect a 25% improvement in DE50 biomarkers values towards WT values, using a small cohort of 3 DMD and 3 WT dogs. 

 The figures allow to see the entire data set for each individual. The raw data set is made available on Figshare, it is well organized and allows tracking at the individual level. In this regard, I would like to credit the authors for mentioning in the body of the article the special cases of the dogs DE50-I2 (Figure 4) and WT-H4 (Figure 5b), outliers for certain values. The occurrence of outliers is often omitted from published data sets, probably to avoid any loss of robustness or strength of conviction. Yet these data are a frequent observation in laboratories, reflecting biological variability, and it is very valuable to find them here.

**Suggestions for improving the article**

1. It would be useful to mention the existence of urinary biomarkers of myonecrosis and to explain why, although they are even less invasive than blood biomarkers, they were not added to this study (e.g. the N-ter fragment of TITIN, see for example Shirakawa *et al*. 2022 ^1^).
2. The authors focused on the myogenic lineage and did not investigate the cellular component of the stem cell niche, in particular the inflammatory component (TNF, IFN, IL-1 or IL-6 pathways), senescence (SASP) or fibrosis (TGFß). Can this restrictive choice be explained and justified?
3. Given that cardiorespiratory distress is fatal in children, it is unfortunate that biomarkers of cardiomyocyte-specific myonecrosis (e.g. cardiac troponin I) were not included. Can the authors discuss this point, even though the title of the article is explicit about targeting skeletal muscle?

*Minor suggestions*

1. Figure 1. I think removing the border of the cells beyond euthanasia of the dogs would be more readable than graying them. We are used to paying attention to the shaded cells while here we actually have to ignore them, it's a bit confusing. If appropriate, the last sentence of the figure's legend can be deleted.
2. Throughout the text, separate numbers and units with a non-breaking space.
3. MYOM3: why did the authors perform the quantification by western blot instead of ELISA? Did they test some of the available kits that did not work? More information on this point would be welcome in the Materials and Methods section.
4. MSTN: the dilutions performed for the WT and DE50-MD dog sera could be specified in the text.
5. Maybe this interesting reference could be added: Grounds *et al*. 2020 ^2^.
6. Third paragraph of the discussion, I guess "in" is missing: "As seen in other animal models of DMD".

Is the work clearly and accurately presented and does it cite the current literature?

Yes

If applicable, is the statistical analysis and its interpretation appropriate?

Yes

Are all the source data underlying the results available to ensure full reproducibility?

Yes

Is the study design appropriate and is the work technically sound?

Yes

Are the conclusions drawn adequately supported by the results?

Yes

Are sufficient details of methods and analysis provided to allow replication by others?

Yes

Reviewer Expertise:

Comparative medical genetics, translational myology, mouse, rat & dog models of neuromuscular disorders

I confirm that I have read this submission and believe that I have an appropriate level of expertise to confirm that it is of an acceptable scientific standard.

## References

[1] : A sandwich ELISA kit reveals marked elevation of titin N-terminal fragment levels in the urine of mdx mice. *Animal Model Exp Med* .5(1) : 10.1002/ame2.12204 48-55 10.1002/ame2.12204 35229992PMC8879618

[2] : Biomarkers for Duchenne muscular dystrophy: myonecrosis, inflammation and oxidative stress. *Disease Models & Mechanisms* .2020;13(2) : 10.1242/dmm.043638 10.1242/dmm.043638 PMC706366932224496

# Response

**Published:** 2022-07-29

**Authors:** Dominique Riddell

**Affiliations:** Royal Veterinary College, London, UK

We thank the reviewer for their kind words, thorough analysis and very encouraging assessment of our work. We agree with the sentiment that showing data for individual animals is very important, as variation between individuals is a common feature of DMD animal models and, of course, DMD patients; we are pleased that you found our dataset organised and easy to interpret.

1. *It would be useful to mention the existence of urinary biomarkers of myonecrosis and to explain why, although they are even less invasive than blood biomarkers, they were not added to this study (e.g. the N-ter fragment of TITIN, see for example Shirakawa et al. 2022* *^1^* *).*

Urinary samples are indeed even less invasively obtained than blood samples (if obtained by free-catch rather than cystocentesis) and based on the findings that elevated TITIN fragments and markers of oxidative damage to urinary proteins are observed in dystrophic individuals (Terrill et al. 2020), urinary biomarkers were of interest to us. However, at present we have not collected urine samples from the DE50-MD dog for this purpose – it is surprisingly challenging to obtain free catch urine samples (on demand) from small dogs in a timely fashion, especially in the youngest animals; further, cystocentesis, which is commonly conducted for this purpose clinically in dogs, is invasive and we felt was best avoided. As the DMD blood biomarker profile is better established and more widely used than the urine profile, we wanted to quantify well known blood markers spanning a range of dystrophic processes for comparison with DMD patients and other animal models. Therefore, after consideration, we concluded that it would be a time-consuming and challenging endeavour to regularly obtain both blood and free-catch urine samples from such a large number of group-housed animals. However, this is potentially something we could explore in future studies. We have added a brief discussion of urinary biomarkers to our introduction.

- *2. The authors focused on the myogenic lineage and did not investigate the cellular component of the stem cell niche, in particular the inflammatory component (TNF, IFN, IL-1 or IL-6 pathways), senescence (SASP) or fibrosis (TGFß). Can this restrictive choice be explained and justified?*

Alongside finding blood-borne biomarkers for our pre-clinical trials, it was also very important to confirm that the DE50-MD model was a good representation of DMD in human patients; we feel that the results from this initial panel of well-known musculoskeletal biomarkers in DMD patient serum convincingly do this. However, we are expanding our investigations to quantify other potential blood-borne biomarkers for other facets of dystrophic pathology. This includes a large panel of inflammatory cytokines (including TNF, IFN, IL1, IL6 and more), as well as biomarkers of cardiac pathology; these results are currently being prepared for incorporation into inflammatory and cardiac phenotype focussed papers.

- 3. *Given that cardiorespiratory distress is fatal in children, it is unfortunate that biomarkers of cardiomyocyte-specific myonecrosis (e.g. cardiac troponin I) were not included. Can the authors discuss this point, even though the title of the article is explicit about targeting skeletal muscle?*

As in DMD patients, the DE50-MD model does develop a cardiac phenotype with increasing age. This includes features characteristic of human DMD dystrophic cardiac pathology (including evidence of increasing ventricular fibrosis and decline in function). However, the DE50-MD cardiac phenotype typically develops beyond 18 months of age, which was the cut-off point for our primary natural history study. An extended cardiac study (to 36 months of age) was performed in a subset of dogs and the findings from this work will form a series of comprehensive papers detailing all of our cardiac-related results. We have chosen to incorporate our cardiac-specific blood-borne biomarker work (which includes cTnI) into this cardiac work, in order to correlate findings from different aspects of this body of work. 

*Minor suggestions*

- *1. Figure 1. I think removing the border of the cells beyond euthanasia of the dogs would be more readable than graying them. We are used to paying attention to the shaded cells while here we actually have to ignore them, it's a bit confusing. If appropriate, the last sentence of the figure's legend can be deleted.*

Thank you for this suggestion; we agree and we have updated this figure.

- *2. Throughout the text, separate numbers and units with a non-breaking space.*

We have made these changes.

- *3. MYOM3: why did the authors perform the quantification by western blot instead of ELISA? Did they test some of the available kits that did not work? More information on this point would be welcome in the Materials and Methods section.*

While there are several MYOM3 ELISA kits available commercially that are reactive for human and mouse samples, we were unable to find any that are developed for or have confirmed cross-reactivity with canine tissue samples. We were inspired to quantify MYOM3 by the 2015 paper by Rouillon *et al*. that we reference in our introduction; this study quantified MYOM3 in the GRMD dog model by western blot using an anti-human MYOM3 antibody. They confirmed that the anti-human MYOM3 antibody detected the same sized bands in human and dog serum, and validated their western blot results with a mass spectrometry approach. We therefore performed western blotting using this same antibody, allowing us to identify MYOM3 in our dog samples with confidence whilst enabling comparison of our DE50-MD results with the GRMD results presented in the aforementioned paper. We have added a sentence to our methods to support our choice of antibody.

- *4. MSTN: the dilutions performed for the WT and DE50-MD dog sera could be specified in the text.*

The final dilution was 1:20, and this has been added to the methods.

- *5. Maybe this interesting reference could be added: Grounds et al. 2020* *^2^* *.* *2. Grounds M, Terrill J, Al-Mshhdani B, Duong M, et al.: Biomarkers for Duchenne muscular dystrophy: myonecrosis, inflammation and oxidative stress.* *Disease Models & Mechanisms* *. 2020;* ***13***** *(2). *

We have cited this excellent review in our discussion of urinary biomarkers.

- *6. Third paragraph of the discussion, I guess “in” is missing: “As seen inother animal models of DMD”.*

Thank you, this has been changed.

# Peer review: Reviewer response for version 1

**DOI:** 10.21956/wellcomeopenres.19235.r47721

**Published:** 2022-01-17

**Authors:** Clare Rusbridge

**Affiliations:** 1 The School of Veterinary Medicine, University of Surrey, Guildford, UK; 2 Wear Referrals, Linnaeus Group, Durham, UK

**Longitudinal assessment of blood-borne musculoskeletal disease biomarkers in the DE50-MD dog model of Duchenne muscular dystrophy**

 This study established if a large animal model is appropriate to study response to a therapeutic intervention. Compared to WT dogs, DE50-MD dogs had substantially higher circulating creatine kinase (CK) activities, myomesin-3 (MYOM3), and the dystromiRs miR-1, miR-133a and miR-206, but significantly lower serum myostatin concentrations. An age-associated pattern, similar to that observed in DMD patients, was seen for CK and MYOM3. The blood biomarker profile closely mimics that of DMD patients, supporting the hypothesis that this DMD model would be suitable for use in pre-clinical trials. The study achieved its hypothesis. Overall, I felt that this was a straightforward question, an appropriate study to answer that question with pertinent results.

 My questions are minor.

1. I would like greater clarification about how much exercise the dogs had. How big are the outside paddocks and is this the only exercise the dogs have? I would also like to know how big the “large pens are” and what it is the range of group size. Also were wild type dogs housed with affected dogs? Myomesin-3 is a biomarker for exercise-induced muscle damage so I would like an indication if the dogs were able to achieve a what would be regarded as a normal exercise level (for a dog).
2. Page 5 – do you mean congenital elbow dysplasia or developmental elbow dysplasia? I suspect the latter.
3. Did you do exercise tolerance tests in the affected dogs. After reading this paper I was left with the question – yes these seem like relevance biomarkers, but do they reflect clinical disease – i.e. is a normalisation of Myomesin-3 likely to result in a phenotypic improvement or were dogs with higher Myomesin-3 phenotypically worse (with the exception of the dysphagic dogs which was phenotypically worse).

Is the work clearly and accurately presented and does it cite the current literature?

Yes

If applicable, is the statistical analysis and its interpretation appropriate?

Yes

Are all the source data underlying the results available to ensure full reproducibility?

Yes

Is the study design appropriate and is the work technically sound?

Yes

Are the conclusions drawn adequately supported by the results?

Yes

Are sufficient details of methods and analysis provided to allow replication by others?

Yes

Reviewer Expertise:

I am a veterinary neurologist and a clinician that specialises in muscle disease. Therefore I am not as competent as many in assessing the validly of the assays used to measure the biomarkers. I am also not an expert statistician.

I confirm that I have read this submission and believe that I have an appropriate level of expertise to confirm that it is of an acceptable scientific standard.

# Response

**Published:** 2022-07-29

**Authors:** Dominique Riddell

**Affiliations:** Royal Veterinary College, London, UK

Thank you for your supportive assessment of our work; we are happy to address the questions raised:

1. *I would like greater clarification about how much exercise the dogs had. How big are the outside paddocks and is this the only exercise the dogs have? I would also like to know how big the “large pens are” and what it is the range of group size. Also were wild type dogs housed with affected dogs? Myomesin-3 is a biomarker for exercise-induced muscle damage so I would like an indication if the dogs were able to achieve a what would be regarded as a normal exercise level (for a dog).*

We have now expanded upon this in our Animal Husbandry section of the Methods. There are 6 outdoor paddocks that are each approximately 100 square metres in size (range 85-115m ^2^), allowing the dogs to move freely and play with toys and each other. The dogs typically have access to the paddocks between 8am and 3pm every day, however this may vary with factors such as very bad weather or a requirement to be kept inside for study-related procedures. The dogs are not walked outside of this biosecure unit area, so are not exercised in addition to having free range daily paddock exercise.

 WT dogs were housed with DE50-MD dogs. Study males were typically housed in groups of 2-3 per indoor kennel, however group size could be up to 5 when in the outdoor paddocks. Groups were established based on compatible social hierarchies. Kennel size varied slightly, but was approximately 3 metres by 1.5 metres for a small kennel and 3 metres by 2.5 metres for a large kennel. Importantly, the ASPA code of practice (Section 2) regarding number of dogs per area of kennels size was followed at all times within the unit. As dysphagia occurred in several DE50-MD study animals, this was closely monitored and any animals with difficulty eating were fed separately before being returned to their group kennel.

 Our team have been conducting longitudinal activity monitoring as part of this natural history study, involving dogs wearing accelerometers for 48 hour periods, objectively to quantify normal daily activity. Preliminary findings suggest that DE50-MD dogs are less active than WT dogs, and that their activity intensity steadily decreases from 3 months of age. We aim in the future to compare activity data for individual dogs with the biomarker profile of their blood samples taken at corresponding timepoints. In addition, we have conducted an exercise study measuring all of the biomarkers described in the current manuscript before and at several timepoints following a 6-minute walk test. This forms part of a larger body of work, optimising and validating the use of this commonly-used exercise tolerance test in the DE50-MD model, and is of sufficient volume as to merit its own dedicated journal article (which is currently in preparation).

1. *Page 5 – do you mean congenital elbow dysplasia or developmental elbow dysplasia? I suspect the latter.*

Thank you for this note. Yes, we agree that developmental elbow dysplasia would be the correct term for this case and have changed the text.

1. *Did you do exercise tolerance tests in the affected dogs. After reading this paper I was left with the question – yes these seem like relevance biomarkers, but do they reflect clinical disease – i.e. is a normalisation of Myomesin-3 likely to result in a phenotypic improvement or were dogs with higher Myomesin-3 phenotypically worse (with the exception of the dysphagic dogs which was phenotypically worse).*

This is an excellent question. In terms of characterisation of clinical disease and correlation with blood biomarkers, this study forms part of a much larger natural history study that assessed a vast range of phenotypic parameters. Measures of exercise tolerance were assessed; in addition to the 6-minute walk test and activity monitoring (described above), muscle physiology protocols measuring muscle force output in response to an electric stimulus in anaesthetised animals were performed as a further objective measure of muscle function. Once each individual component of the study is fully analysed we will have the opportunity to see if the (relatively easy to obtain) blood biomarker profile correlates with any other particular phenotypic characteristics of the model. Further, all of the biomarkers identified in this study can be measured in pre-clinical trials using this model of DMD, so that we would be able to confirm whether or not they show utility as biomarkers of phenotypic improvement following therapy. We have added several sentences in the penultimate paragraph of our discussion that consider whether the blood-borne biomarkers might reflect specific aspects of clinical disease in the dogs.
